# Supplementary material for: Development and pilot application of a point-of-need molecular xenomonitoring protocol for tsetse (Glossina sp.) in a low-resource setting
Source: PLoS Negl Trop Dis. 2026 Mar 23;20(3):e0013706. doi: 10.1371/journal.pntd.0013706 (PMC13035148; doi:10.1371/journal.pntd.0013706)
Supplement: S1 Table — TM1 = TE buffer, TM2 = lysis buffer and TM3 = alkaline extraction. SE = standard error. (PDF) [file pntd.0013706.s003.pdf]

Replicate number	Test method			Gold standard
	**TM1**	**TM2**	**TM3**	**Qiagen**
**1**	28.52		20.11	17.52
**2**	**-**	23.95	21.66	17.50
**3**	**-**	21.30	19.16	18.30
**4**	**-**	21.71	23.16	18.77
**5**	37.02	21.65	20.68	18.29
**6**	**-**	23.12	19.11	17.39
**7**	29.52	21.57	21.01	21.63
**8**	**-**	27.51	22.15	17.15
**9**	**-**	21.98	23.10	16.76
**Mean Cq**	**31.69**	**22.85**	**21.13**	**18.15**
**Cq SE**	**2.68**	**0.74**	**0.51**	**0.48**

S1 Table: A table displaying Cq values obtained from *Wigglesworthia*-qPCR (S1 Figure) screening of nine insectary-reared *G. m. morsitans* DNA extracted using three field extraction methods (TM1-3), compared against a gold standard extraction method (QIAGEN DNeasy Blood and Tissue; S1 Figure). TM1 = TE buffer, TM2 = lysis buffer and TM3 = alkaline extraction. SE = standard error.
