# Supplementary material for: Development and pilot application of a point-of-need molecular xenomonitoring protocol for tsetse (Glossina sp.) in a low-resource setting
Source: PLoS Negl Trop Dis. 2026 Mar 23;20(3):e0013706. doi: 10.1371/journal.pntd.0013706 (PMC13035148; doi:10.1371/journal.pntd.0013706)
Supplement: S2 Table — SD = standard deviation, PP = proportion positive. (PDF) [file pntd.0013706.s004.pdf]

a) *T. brucei* (TBR)

DNA conc (copies/µL)	$1.88x10^{0}$	$1.88x10^{-1}$	$1.88x10^{-2}$	$1.88x10^{-3}$	$1.88x10^{-4}$
**Mean Cq**	26.59	29.78	35.40	NA	NA
**SD**	1.40	0.18	2.22	NA	NA
**Amplification**	4	4	4	0	0
**PP (%)**	100%	100%	100%	0%	0%

b) *T. congolense* Forest (TCF)

DNA conc (copies/µL)	$1.22x10^{0}$	$1.22x10^{-1}$	$1.22x10^{-2}$	$1.22x10^{-3}$	$1.22x10^{-4}$
**Mean Cq**	29.04	31.40	34.86	NA	NA
**SD**	0.33	1.66	1.84	NA	NA
**Amplification**	4	4	2	0	0
**PP (%)**	100%	100%	50%	0%	0%

c) *T. vivax* (TVX)

DNA conc (copies/µL)	$1.97x10^{1}$	$1.97x10^{0}$	$1.97x10^{-1}$	$1.97x10^{-2}$	$1.97x10^{-3}$
**Mean Cq**	25.08	27.87	31.64	NA	NA
**SD**	1.06	0.31	1.01	NA	NA
**Amplification (/4)**	4	4	2	0	0
**PP (%)**	100%	100%	50%	0%	0%

S2 Table: Tables displaying analytical sensitivity limit-of-detection (LOD) results for the optimised dry-format Multi-Tryp qPCR against three trypanosome targets *T. brucei* s-l (a), *T. congolense* Forest (b) and *T. vivax* (c). SD = standard deviation, PP = proportion positive.
