# Supplementary material for: Development and pilot application of a point-of-need molecular xenomonitoring protocol for tsetse (Glossina sp.) in a low-resource setting
Source: PLoS Negl Trop Dis. 2026 Mar 23;20(3):e0013706. doi: 10.1371/journal.pntd.0013706 (PMC13035148; doi:10.1371/journal.pntd.0013706)
Supplement: S3 Table — (PDF) [file pntd.0013706.s005.pdf]

a)

Species	Sub-species	Strain	DNA conc.	Target (Y/N)	Amplification (total = 3)	Mean Cq.
T. brucei	*brucei*	AnTat 1.1	1ng/µL	Y	3	11.81
T. brucei	*gambiense*	ELIANE	1ng/µL	Y	3	17.45
T. brucei	*rhodesiense*	Z3	1ng/µL	Y	3	10.72
T. congolense	Savannah	IL3000	1ng/µL	N	0	-
T. congolense	Forest	ANR3	10fg/µL	N	0	-
T. congolense	Kilifi	WG84	1ng/µL	N	0	-
T. simiae		TV008	1ng/µL	N	0	-
T. simiae	Tsavo	114	1ng/µL	N	0	-
T. vivax		Y486	1ng/uL	N	0	-
G. f. fuscipes **(West Nile) +** ***W. glossinidia*** **composite**			Unknown	N	0	-

b)

Species	Sub-species	Strain	DNA conc.	Target (Y/N)	Amplification (total = 3)	Mean Cq.
T. brucei	*brucei*	AnTat 1.1	1ng/µL	N	0	-
T. brucei	*gambiense*	ELIANE	1ng/µL	N	0	-
T. brucei	*rhodesiense*	Z3	1ng/µL	N	0	-
T. congolense	Savannah	IL3000	1ng/µL	N	0	-
T. congolense	Forest	ANR3	10fg/µL	Y	3	25.71
T. congolense	Kilifi	WG84	1ng/µL	N	0	-
T. simiae		TV008	1ng/µL	N	1	38.27
T. simiae	Tsavo	114	1ng/µL	N	0	-
T. vivax		Y486	1ng/uL	N	0	-
G. f. fuscipes **(West Nile) +** ***W. glossinidia*** **composite**			Unknown	N	0	-

c)

Species	Sub-species	Strain	DNA conc.	Target (Y/N)	Amplification (total = 3)	Mean Cq.
T. brucei	*brucei*	AnTat 1.1	1ng/µL	N	0	-
T. brucei	*gambiense*	ELIANE	1ng/µL	N	0	-
T. brucei	*rhodesiense*	Z3	1ng/µL	N	0	-
T. congolense	Savannah	IL3000	1ng/µL	N	0	-
T. congolense	Forest	ANR3	10fg/µL	N	0	-
T. congolense	Kilifi	WG84	1ng/µL	N	0	-
T. simiae		TV008	1ng/µL	N	0	-
T. simiae	Tsavo	114	1ng/µL	N	0	-
T. vivax		Y486	1ng/uL	Y	3	12.56
G. f. fuscipes **(West Nile) +** ***W. glossinidia*** **composite**			Unknown	N	0	-

d)

Species	Sub-species	Strain	DNA conc.	Target (Y/N)	Amplification (total = 3)	Mean Cq.
T. brucei	*brucei*	AnTat 1.1	1ng/µL	N	0	-
T. brucei	*gambiense*	ELIANE	1ng/µL	N	0	-
T. brucei	*rhodesiense*	Z3	1ng/µL	N	0	-
T. congolense	Savannah	IL3000	1ng/µL	N	0	-
T. congolense	Forest	ANR3	10fg/µL	N	0	-
T. congolense	Kilifi	WG84	1ng/µL	N	0	-
T. simiae		TV008	1ng/µL	N	0	-
T. simiae	Tsavo	114	1ng/µL	N	0	-
T. vivax		Y486	1ng/uL	N	0	-
G. f. fuscipes **(West Nile) +** ***W. glossinidia*** **composite**			Unknown	Y	3	19.27

S3 Table: Tables displaying optimised dry-format Multi-Tryp qPCR analytical specificity testing results across the four targets; TBR - ***T. brucei*** **s-l (a), TCF-** ***T. congolense*** **Forest (b), TVX -** ***T. vivax*** **(c) and UGWigg –** ***W. glossinidia*** **(d).**
