# Supplementary figures and images for: Development and pilot application of a point-of-need molecular xenomonitoring protocol for tsetse (Glossina sp.) in a low-resource setting
Source: PLoS Negl Trop Dis. 2026 Mar 23;20(3):e0013706. doi: 10.1371/journal.pntd.0013706 (PMC13035148; doi:10.1371/journal.pntd.0013706)

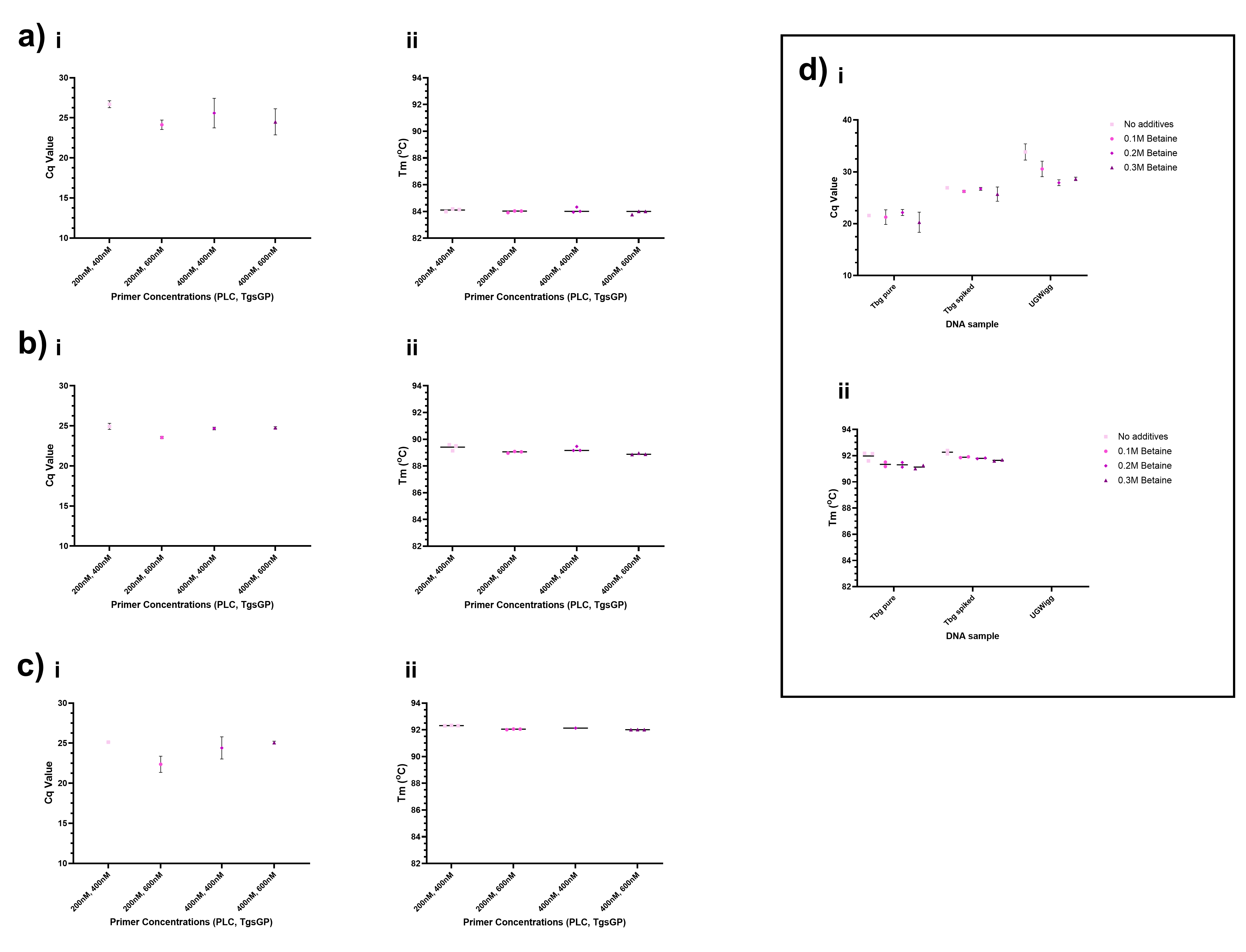

Supplement: S2 Fig — DNA screened was T. b. brucei, T. b. gambiense and T. b. rhodesiense at 10pg/µL. (d) displays results of HAT-HRM betaine optimisation experiments in pure T. b. gambiense (Tbg) DNA (100pg/µL), Tbg DNA spiked into G. f. fuscipes composite DNA (10pg/µL Tbg) and pure G. f. fuscipes composite DNA. Error bars represent standard error, horizontal bars represent the mean. (TIF) [file pntd.0013706.s006.tif]

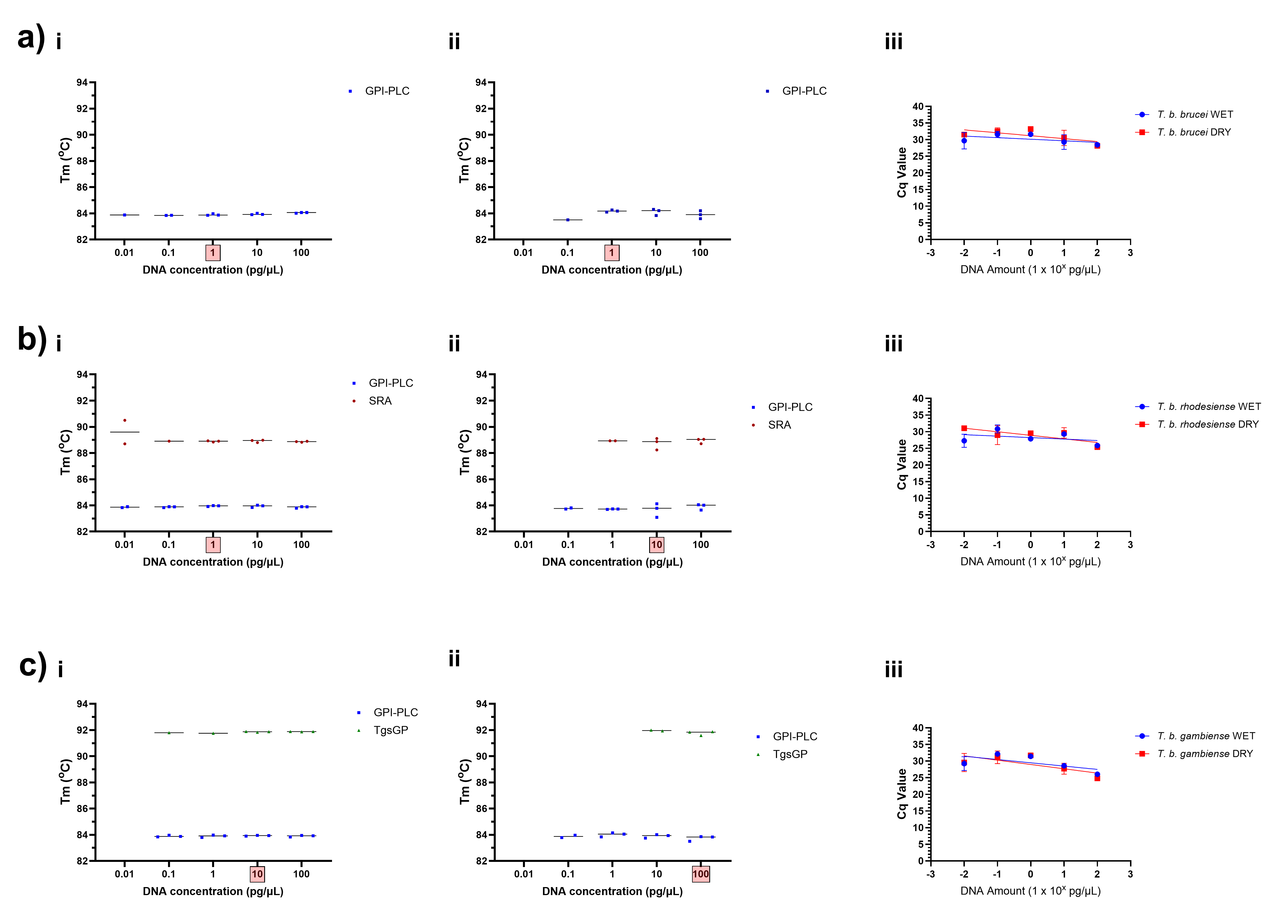

Supplement: S3 Fig — DNA: (a) T. b. brucei (PLC), (b) T. b. rhodesiense (PLC + SRA) and (c) T. b. gambiense (PLC + TgsGP). In charts (i) and (ii), melt temperatures (Tm) for each amplified target product (PLC, blue square; SRA, red circle; TgsGP, green triangle) are plotted. Horizontal bars represent the mean, red shaded value on x-axis represents the 95% limit-of detection. In charts (iii), HAT-HRM mean Cq values and standard curves are plotted for wet (blue, circle symbol) and dry (red, square symbol) format reactions. Error bars represent standard error. (TIF) [file pntd.0013706.s007.tif]
