# Supplementary material for: Development and pilot application of a point-of-need molecular xenomonitoring protocol for tsetse (Glossina sp.) in a low-resource setting
Source: PLoS Negl Trop Dis. 2026 Mar 23;20(3):e0013706. doi: 10.1371/journal.pntd.0013706 (PMC13035148; doi:10.1371/journal.pntd.0013706)
Supplement: S4 Fig — All samples (n = 8) amplified Wigglesworthia target DNA (A) and one sample tested positive for T. brucei s-l DNA (B). The melt profile resulting from HAT-HRM screening (C) of this sample purple trace) and NTC (red trace) showed that the sample is likely to be T. b. brucei based on a single melt peak at 82.89oC. (PDF) [file pntd.0013706.s008.pdf]

a) *Wigglesworthia glossinidia*

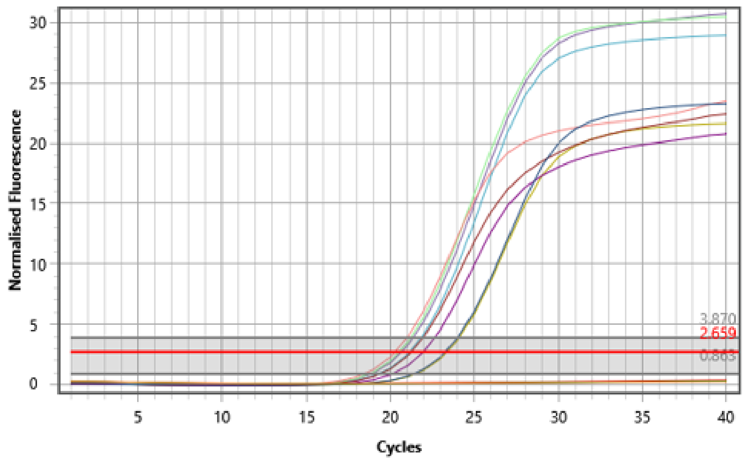

b) *T. brucei* s-l

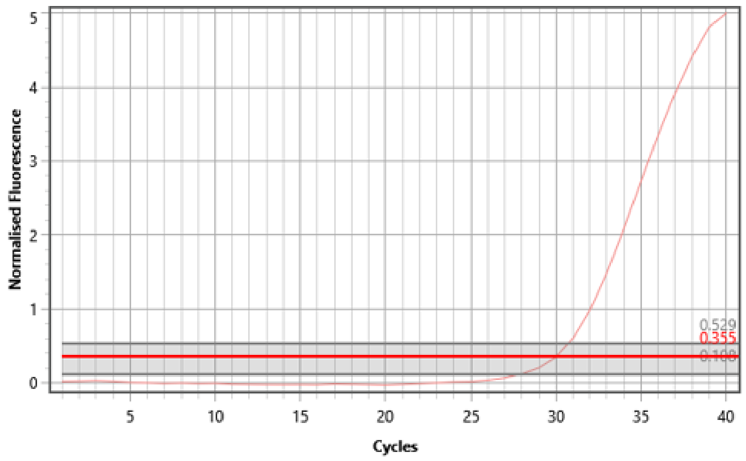

c) HAT-HRM

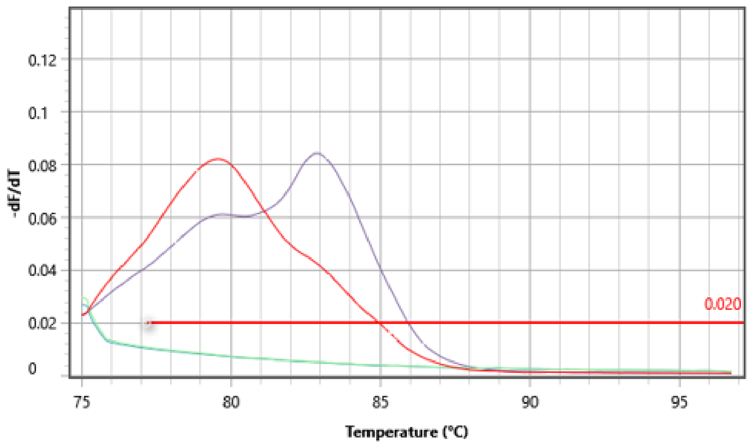

S4 Figure: Images showing amplification traces for UGWigg (A) and TBR (B) targets as part of Multi-Tryp qPCR screening of eight samples, two negative extraction controls and one negative template control (NTC). All samples (n=8) amplified *Wigglesworthia* target DNA (A) and one sample tested positive for *T. brucei* s-l DNA (B). The melt profile resulting from HAT-HRM screening (C) of this sample purple trace) and NTC (red trace) showed that the sample is likely to be *T. b. brucei* based on a single melt peak at 82.89°C.
